# The Mini-Symposium on Global Child Health: Serving the Children of the World

**DOI:** 10.1038/s41390-023-02570-w

**Published:** 2023-04-04

**Authors:** Cristina Scutariu, Davide Bilardi, Francis I. Ayomoh, Charles C. Roehr

**Affiliations:** 1 Department of Experimental Psychology, Medical Sciences Division, University of Oxford, Oxford, Oxfordshire OX2 6GG UK; 2 Centre for Tropical Medicine and Global Health, University of Oxford, Oxford, Oxfordshire OX3 7LG UK; 3 KEMRI-Wellcome Trust Research Programme, P.O.Box 230-80108, Kilifi, Kenya; 4 Nuffield Department of Primary Care Health Sciences, University of Oxford, Oxford, Oxfordshire OX2 6GG UK; 5 National Perinatal Epidemiology Unit, Nuffield Department of Population Health, Medical Sciences, Division, University of Oxford, Oxford, Oxfordshire OX3 7LF UK; 6 Newborn Services, Southmead Hospital, North Bristol Trust, Southmead Road, Bristol, BS10 5NB UK; 7 European Society for Paediatric Research, Rue des Sablières 5, 1242 Satigny, Geneva, Switzerland

## Introduction

The Sustainable Development Goals of the United Nations highlight the importance of reducing the mortality of infants and children under 5 years.^1^ Indeed, since the 1990s improvements were made, decreasing mortality rates by 58%.^2^ However, this decrease does not accurately reflect the situation in low and middle-income countries (LMICs). To date, regions in sub-Saharan Africa still have mortality rates 19 times higher compared to those of high-income countries (HICs).^2^ These findings are a strong call for action to (1) assess factors driving trends in child mortality rates in LMICs, and (2) identify strategies and/or required partnerships to tackle identified issues.

The “Mini-Symposium on Global Child Health: Serving the Children of the World” emerged as an academic response to the above-named challenges. Developed in partnership with the student-led Oxford Global Health and Care Systems Society (OGHCS), the European Society for Paediatric Research (ESPR), The Global Health Network of the University of Oxford, and Academic Paediatrics Association for Great Britain and Ireland, the Mini-Symposium took place on the 7th April 2022 at Green Templeton College (GTC), University of Oxford, UK.

The symposium provided a platform for discussion between experts and emerging leaders and sought to explore potential avenues in the global effort to improve Global Child Health. Importantly, the event emphasised the importance of wider, interdisciplinary partnerships in neonatal and paediatric research aiming to benefit Child Health globally. As such, innovative approaches in neonatal and paediatric research were highlighted throughout three Chaired Sessions, each focusing on specific points of action (Table 1).

**Table 1:** Mini-Symposium on Child Global Health day sessions.

Programme and content	Speakers
**Session 1—Global Children Health: examining the current research.** **Aim**: to examine how current research has tackled challenges in paediatric healthcare in LMICs. **Session chair**: Prof. Charles Roehr	
Maternal and perinatal Health Research Collaboration in India	Associate Prof. Manisha Nair
Established Perinatal Therapies—A need for further evaluation and research	Prof. Cheah Fook Choe
Research in neonatal resuscitation from a global perspective	Prof. Maximo Vento
**Session 2—Global Child Health: The challenge of giving voice to the children** **Aim:** to address the need to actively incorporate the voices/input of children in research **Session chair:** Dr. Davide Bilardi	
Antibiotic prescribing and infection in paediatrics from a global health perspective	Prof. Esmita Charani
Challenge or opportunity? Utilising the voices of children to shape paediatric research	Miss Mercy Shibemba
**“Sun and Petals”—a successful story in Global Child Health**	Introduction to the award-winning documentary by Dr. Davide Bilardi
**Session 3—A strategic vision: looking at the future of Global Child Health** **Aim:** to present and examine strategies in Global Health that foster local and international collaborations in their quest to address challenges in healthcare systems **Session chair**: Prof. Trudie Lang	
The dynamic evolution of paediatric infectious diseases research: the essential role of global collaborations	Prof. Carlo Giaquinto
Embedding research into routine settings—ethnography to pragmatic trials, policy linkages and capacity building	Prof. Mike English
**Session 4—Round table: research needs in global paediatrics** **Co-moderation**: Prof. Charles Roehr and Prof. Trudie Lang	
**Closing Session: Poster Competition**	Presentation of the Poster Competition results. Future initiatives of the student-led OGHCS (Mr. Francis Ayomoh)

The Mini-Symposium hosted three chaired sessions, each focusing on specific points of action and a round table with all speakers as panellists.

## The first session—examining the current research

In the first session, speakers discussed how current research has identified or tackled challenges in paediatric healthcare in LMICs. Associate Professor Dr. Manisha Nair, MRC Career Development Fellow within Oxford Population Health, emphasised that maternal well-being is tightly linked to child health, and discussed how research collaborations drive our understanding of causes of mortality. The Maternal and perinatal Health Research collaboration (MaatHRI), involving 14 hospitals across 4 states in India, was presented as an example medium for large epidemiological studies.^3^ MaatHRI emerged in response to India showing the second highest number of maternal deaths globally and a high number of medical complications during pregnancy,^3^ highlighting the need to understand regional drivers of mortality rates.

Despite the identification of mortality risk factors, establishing effective Perinatal Therapies in resource-constrained settings such as LMICs may be challenging. Professor Dr. Cheah Fook Choe, Head and Professor of Paediatrics at the University Kebangsaan Malaysia, discussed how effective therapies in HICs may have differential, negative outcomes in LMICs. Specifically, the case of antenatal steroids used to accelerate lung maturation following preterm labour in HICs, such as dexamethasone,^4^ was highlighted. Population differences and healthcare possibilities may account for such results and should be taken into consideration for in the design of future clinical trials.

Professor Maximo Vento, Professor of Paediatrics and Neonatology at the University and Polytechnic Hospital La Fe, suggested similar disparities between HICs and LMICs in research on neonatal resuscitation. Indeed, the recommended treatment for neonatal encephalopathy in HICs, therapeutic hypothermia (TH), was suggested to be associated with increased mortality in LMICs in the Helix study.^5^ More positive results may however be achieved with the application of basic science in clinical settings: room air oxygen levels were later associated with better outcomes and may be more easily established in limited resource settings.^6^ Nevertheless, poorer healthcare system infrastructure may still affect LMICs and the efficiency of TH beyond oxygen levels, highlighting the need to comprehensively assess resources and requirements in LMICs.

## The second session—the challenge of giving voice to the children

The focus of the second session of the Mini-Symposium was to analyse the challenge of incorporating vulnerable communities and children in research which aims to benefit them. Dr. Esmita Charani, a leading researcher at Imperial College London, used antibiotic usage patterns to exemplify the need to refocus efforts in research to include paediatric populations. Specifically, whilst preventing antimicrobial resistance is a top priority for the current medical practice, several challenges e.g. the lack of agreement on standardised metrics for monitoring paediatric antibiotic consumption prevent the implementation of training and stewardship programmes, which foster appropriate prescription behaviours.^7^ Such findings warrant the need for inclusion and further study of paediatric participants in clinical trials.

Miss Mercy Shibemba, an award-winning activist, followed and used her story of growing up with HIV to educate on the benefits of including children’s perspectives in healthcare decisions. She presented lessons from the Youth Trial Boards,^8^ groups established to enable children and young people experiencing a health condition to participate in the decision-making of relevant clinical trials. The model is currently running in South Africa, Uganda, Zimbabwe, and the UK, and offers training and support for advocates, such as simplifying consent forms, to ensure their contribution at all stages of research.^8^

## Third session—a strategic vision: looking at the future of Global Child Health

The opportunities provided by collaborations, and the benefits of strategic involvement in research were explored in the third session. The contributions of Penta, a scientific network dedicated to paediatric research, were presented by its Director Professor Carlo Giaquinto, also part of the University of Padova. Prof Giaquinto presented the extensive experience of the Penta network in leading strategic collaborations in paediatric research giving an overview of the key areas Global Child Health research should focus on. Among other strategic studies, he presented as an example the Odyssey trial that compared the efficacy of standard HIV treatment in children with new paediatric formulations of antiretroviral therapies bringing together institutions based in LMICs and HICs in a successful partnership.^9^ Essentially, the value of a strategic collaborative approach was highlighted for its potential to conquer persistent and emerging global challenges in child health.

A strategy to integrate the national healthcare community to drive research improvements was next pointed out by Professor Mike English of The University of Oxford. Prof English’ talk outlined how by embedding research into routine settings, such as hospitals, the lack of patient health records which affects LMICs can be approached. The creation of “learning health systems”, which use real world data from hospital records or trial registries, was achieved through the formation of the 2013 Kenyan Clinical Information Network, which by 2020 included 22 hospitals.^10^ The network provides training in various aspects, including evidence-based guideline development, aiming to examine important outcomes of hospitalisation.^10^ These strategies, harnessing the power of data collection, have highlighted that 5–13-year-olds contribute to 20% of all pneumonia-related admissions.^10^ Furthermore, the identification of factors signalling poor outcomes also fuelled discussions on adapting WHO guidance, highlighting the advantages and impact of such networks.

## Closing session: the poster competition

Outside the speaker sessions, the poster competition of the symposium provided early career researchers with a chance to present their work, and thus served as a forum for the presentation of new, original research in the field of Paediatrics. Affirming the success of the initiative, the poster competition received abstract submissions from around the world. The symposium’s Scientific Panel, and the audience on the day, voted for one winning poster each. Dr. Cervantée Wild, a New Zealand Health Research Council Fellow at GTC, was the winner of the Scientific Panel vote for her poster titled “Five-year follow-up of a family-based multidisciplinary programme for children with weight issues: a post-randomised clinical trial analysis”. The participants’ vote selected Lisha Jeena, a DPhil student in Clinical Medicine at The University of Oxford for the poster titled “The effect of HIV-associated chronic inflammation on musculoskeletal health among peripubertal children in Zimbabwe”. Both winners received financial support to attend the European Academy of Paediatric Societies 2022 international conference.

The “Mini-Symposium on Global Child Health: Serving the Children of the World” was a meeting point for world-class speakers and students interested in topics spanning the areas of perinatal medicine and adolescent health, with more than 80 participants attending the event in person and 20 attending remotely. The event succeeded in paving the way for new collaborations, for emerging researchers and more senior academics alike. Moving forward, given the success of the Mini-Symposium in terms of attendance and feedback from attendees, there is interest in expanding the initiative to a recurring, annual event aiming to highlight advances in Global Child Health research.
